# Translational sensitivity of the *Escherichia coli* genome to fluctuating tRNA availability

**DOI:** 10.1093/nar/gkt602

**Published:** 2013-07-10

**Authors:** Sibylle E. Wohlgemuth, Thomas E. Gorochowski, Johannes A. Roubos

**Affiliations:** DSM Biotechnology Center, P.O. Box 1, 2600 MA Delft, The Netherlands

## Abstract

The synthesis of protein from messenger RNA during translation is a highly dynamic process that plays a key role in controlling the efficiency and fidelity of genome-wide protein expression. The availability of aminoacylated transfer RNA (tRNA) is a major factor influencing the speed of ribosomal movement, which depending on codon choices, varies considerably along a transcript. Furthermore, it has been shown experimentally that tRNA availability can vary significantly under different growth and stress conditions, offering the cell a way to adapt translational dynamics across the genome. Existing models of translation have neglected fluctuations of tRNA pools, instead assuming fixed tRNA availabilities over time. This has lead to an incomplete understanding of this process. Here, we show for the entire *Escherichia coli* genome how and to what extent translational speed profiles, which capture local aspects of translational elongation, respond to measured shifts in tRNA availability. We find that translational profiles across the genome are affected to differing degrees, with genes that are essential or related to fundamental processes such as translation, being more robust than those linked to regulation. Furthermore, we reveal how fluctuating tRNA availability influences profiles of specific sequences known to play a significant role in translational control of gene expression.

## INTRODUCTION

Although protein translation is one of the most important cellular processes during bacterial replication and growth, dynamics of this process are still barely understood. The translational dynamics that take place when synthesizing protein from a messenger RNA (mRNA) transcript have been shown to highly influence the quantity and, in some cases, the quality of the resultant protein (1–6). Underlying mechanisms are as follows: (i) the speed of initiation and complex formation of ribosomes at a transcript (7); (ii) sequence features of the mRNA like codon usage, secondary structures, GC content (2–4,8–10); and (iii) availability of resources such as transfer RNA (tRNA) pools (11,12). In addition, there is growing realization that post-transcriptional modifications of tRNAs by uridine methyl-transferases at wobble position 34 also play an important role in both the recognition and sensitivity of particular codon-anticodon pairings (13,14). All of these factors can affect the speed at which a ribosome can join and move along a transcript. Furthermore, pausing and premature termination of ribosomes owing to crowding, rare codon usage (3) and the possible need for co-translational pausing to ensure correct folding of a resultant protein (4–6,10,15) lead to variability in this process. A better understanding of the contribution that these mechanisms have on protein expression is essential to provide a clearer picture of how these features have evolved and become used for regulation purposes by organisms and additionally to enable the improved design of bioengineered systems where protein synthesis plays an important role, e.g. recombinant protein production.

To date, the majority of focus in this area has been on the translational initiation step (7) and codon usage within protein-coding regions (16). However, the transient nature of translation, namely, the sequential concatenation of amino acids, is known to result in a non-uniform protein synthesis rate along an mRNA (15,17). Several forms of model have been proposed to capture this dynamic process. Two of the most common types are stochastic models based on the Totally Asymmetric Simple Exclusion Process (18,19) and deterministic models based on codon adaptation to available tRNA pools (9,10,20). For both type of models, two main assumptions are made: (i) the rate of synthesis at a particular codon is proportional to the concentration of its cognate tRNAs, taking into consideration the sensitivity of possible wobble pairings, and (ii) tRNA concentrations, and therefore codon translation rates, remain fixed throughout a simulation. However, experimental measurements of tRNA concentrations and their charged fractions under stressful conditions have shown that tRNA availability can significantly vary between conditions and over time (11,12), calling into question the validity of these models under such scenarios.

**Figure 1.**
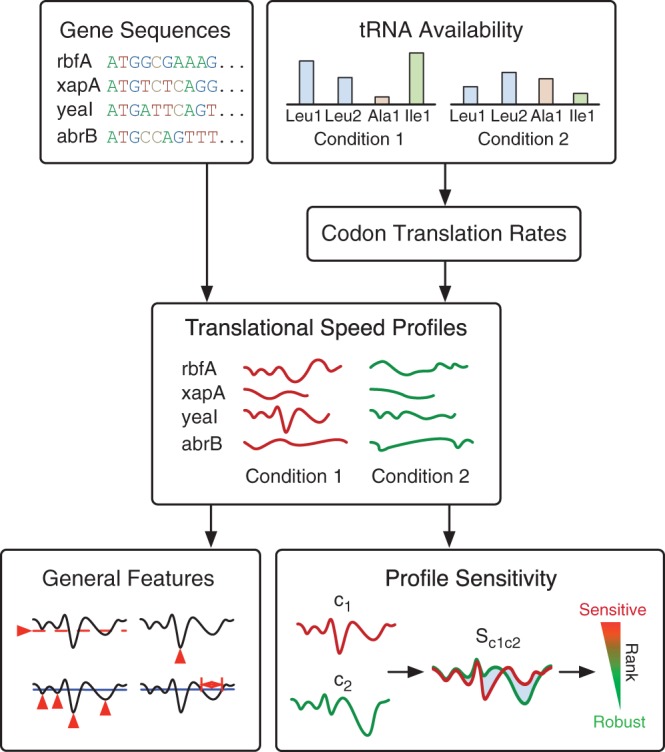
Overview of the workflow used to estimate translational profiles and analyze general features and sensitivities. The main inputs are tRNA availabilities (concentrations and charged fractions) and the gene sequences to be analyzed. In this study, several different sets of tRNA availabilities are used. From these, codon translation rates are calculated and translational profiles generated for each sequence. Profiles are then analyzed in isolation by looking at general features that can be further explored in terms of genome-wide distributions and by comparing changes to profile shapes owing to varying tRNA availability under different conditions (sensitivity analysis).

With the aim to understand the influence that such fluctuations can have on translational dynamics, we developed a generalized computational workflow (Figure 1) to estimate the translational speed at individual codons and generated translational speed profiles for every transcript in the *Escherichia coli* genome. These were based on experimentally measured tRNA concentrations and charging. Unlike previous models, we relaxed the constraint of fixed translation rates and allowed for observed changes in tRNA availability under differing conditions to affect translational speeds of codons.

Our model was based on that of Zhang *et al.* (10), which can efficiently compute translational profiles owing to deterministic rates for each codon. This was essential, given the large number of profiles that needed to be generated for each of the different tRNA availabilities. Furthermore, because our approach focused specifically on the local translational rate along the mRNA, we did not require additional information such as initiation and termination rates not known for most transcripts yet necessary for many alternative modeling approaches (9,19).

Using this tool, we investigated how features of the translational profiles varied under differing conditions and developed a method to assess the sensitivity of transcripts to shifts in the tRNA availability.

## MATERIALS AND METHODS

### Sequence and tRNA availability data sets

We applied the described methods to all *E. coli* K-12 coding sequences (GenBank accession number: NC_000913.2). Availability of tRNAs was obtained from two experimental data sets: (i) tRNA concentrations measured at different growth rates (12)—in this data set, the concentrations for Gly1 and Gly2 as well as the concentrations for Ile1 and Ile2 are treated collectively, and to obtain the individual concentrations, the values were split according to the ratio of the gene copy numbers for the two isoacceptors (Gly1:Gly2 = 1:1, Ile1:Ile2 = 3:1) and (ii) tRNA charging values at different times after leucine starvation (11)—absolute concentrations were obtained by multiplying the charging values with the concentrations at a growth rate of 2.5 doublings per hour, taken from (12).

### Codon translation rates

Our method to estimate codon translation rates from tRNA concentrations is based on the calculation of the tRNA adaption index (21). The adaptiveness value 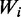 for each codon *i* that is recognized by 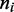 tRNA isoacceptors is defined as
(1)
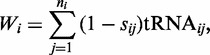

where 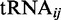 denotes the concentration of the *j*th tRNA that recognizes the *i*th codon, 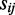 is a selective constraint on the efficiency of the codon-anticodon coupling, with each value adopted from (21) and related to one wobble pairing (Supplementary Table S1). The codon-anticodon recognition pattern is defined according to Crick’s Wobble rules (22), with codons grouped into blocks of four elements reflecting all possible interactions (Supplementary Figure S1). Formulas for the calculation of adaptiveness values, 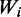, for each element *n* in a block are defined in Supplementary Table S2 and taken from (21). As an exception, the adaptiveness value for the AUA codon is calculated as 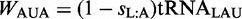.

The s-value for recognition of the CGA codon by the ACG anticodon is high resulting in a low rate for this codon (about two orders of magnitude smaller than the next highest rate). This rate is sensitive to this particular s-value, and owing to its extremely low value, it dominates the analysis. This has been recognized previously (10,20) and therefore we set the s-value for this interaction to 0.9172 as used by (10), which better matches experimental data.

To obtain the rate 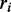 for each type of codon *i*, the adaptiveness values are normalized by the sum of the values over all codon types, giving the probability for the coupling of a matching tRNA (20). To account for variations in total tRNA pool size between data sets, this value is multiplied by the sum of all tRNA concentrations in the pools
(2)
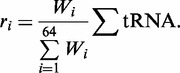



### Translational speed profiles

For the generation of a translational speed profile along a coding sequence, each codon *i* within the sequence is assigned a translation time 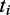. We assume that the translational rate of a codon is proportional to the available charged cognate tRNA (10), which gives an expected codon translation time of 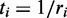 (20). Smoothed translational profiles were then generated by averaging the codon translation times over a centered sliding window of 19 codons. This window size is adopted from (10) to incorporate possible local effects of the mRNA sequence and is based on the ribosomal footprint.

### Translational sensitivity

To investigate the sensitivity of profiles to shifts in tRNA availability, comparisons were made between profiles before and after a shift. For each data set, we designated a reference profile, ‘c1’, to capture the profile under standard conditions: either 2.5 doublings per hour for growth rate data or t = 0 min for leucine starvation. Profiles for comparison, ‘c2’, were then generated for the remaining conditions (growth rates of 0.4, 0.7, 1.07, 1.6 doublings per hour and t = 2, 7, 17, 32 min after leucine starvation). A sensitivity measure was defined 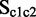 as the sum of the absolute differences between the translation times of each codon *j* within the smoothed profiles ‘c1’ and ‘c2’. The resulting absolute sensitivity was then normalized by the length *l* of the profile to facilitate comparisons between genes of differing lengths
(3)
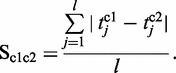



### Threshold calculation

To analyze general features of a translational profile in relation to characteristics of the entire genome, we used a threshold that captured the average codon speed of all smoothed translational profiles under the corresponding reference condition. A single threshold was used for each data set to enable a fair comparison between conditions. This threshold enabled us to identify regions in a profile that displayed slower translational rates than the rest of the genome, with deep minima highlighting possible points at which translational pausing might occur (10). The threshold for the growth rate data set was 0.2191 (from 2.5 doublings per hour), and for leucine starvation, it was 0.2577 (from t = 0 min).

### General profile features

To capture general features of the translational profiles, we calculated (i) the average speed at which a sequence is translated, corresponding to the arithmetic mean across the smoothed profile; (ii) the slowest point, defined as the longest translation time within the smoothed profile; (iii) the drop count, given by the number of times the smoothed profile drops below the threshold (if the entire profile is below the threshold, the drop count is set to 1); and (iv) the maximal drop length, calculated as the width (in numbers of codons) of a profiles longest region below the threshold (profiles that entirely lie below the threshold are not included in the analysis of this feature).

### Functional enrichment analysis

Lists of UniProt accession numbers were generated for the 10% most and 10% least sensitive gene sequences under each condition of interest. Pseudo genes and nucleotide sequences were excluded from these lists. Functional enrichment was then performed using the AmiGO term enrichment tool (23). All protein-coding sequences from *E. coli* were selected as the background set for comparison, and other parameters took default values: ‘use IAEs in calculation’ = yes, ‘maximum p-value’ = 0.01, ‘minimum number of gene products’ = 2.

### Genome-wide visualization

Features of the *E. coli*-coding sequences were visualized for the whole genome using the Circos tool (24). Pseudo genes were excluded from this analysis. To capture general features of genes clustered on the genome (e.g. owing to operon structures), the genome was divided into 200 stretches of 23 198 bp and a heat map produced of averages over these stretches.

## RESULTS

### Codon translational speeds vary between conditions

To investigate how known changes in tRNA availability influence translational elongation in *E. coli*, we used two published experimental data sets (‘Materials and Methods’ section). The first data set provides tRNA concentrations in *E. coli* growing at rates of 2.5 (standard), 1.6, 1.07, 0.7 and 0.4 doublings per hour (12). The second data set records tRNA concentrations and charging levels in *E. coli* before (t = 0 min) and at four time points (t = 2, 7, 17, 32 min) after leucine starvation (11). From these concentrations and charged fractions, we were able to estimate codon translation rates using a method based on the tRNA adaptation index (21). This approach was chosen because it has been shown to fit reasonably well to experimental data (9). It also provides a standardized way of applying our approach to alternative organisms where experimentally measured tRNA concentrations may not yet be available (i.e. only tRNA gene copy numbers are required to estimate standard rates).

**Figure 2.**
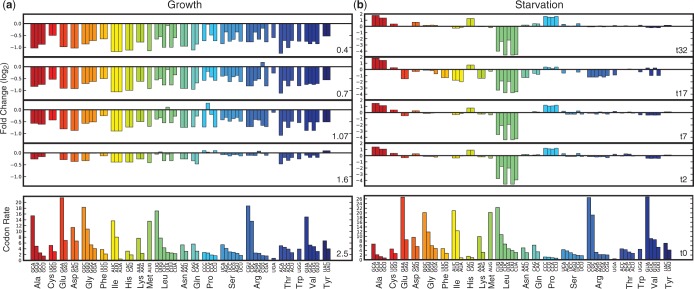
Estimated codon translation rates vary owing to differences in tRNA concentrations and charging. For both (**a**) growth rate data and (**b**) leucine starvation data, the absolute values of the codon translation rates at standard reference conditions are shown on the bottom subplots (growth rate of 2.5 doublings per hour and t = 0 for leucine starvation). Codons are colored according to the amino acid they code for. The top four subplots show the fold change in the rates, *r*, under each condition compared with the reference condition, 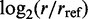. During leucine starvation, both decreases and increases in codon rates are observed (e.g. codons for proline, histidine, alanine).

The speed at which a particular codon is translated depends on the availability of the cognate tRNAs. Thus, the described shifts in tRNA abundance mean that local translation rates of codons can vary under different conditions (Figure 2).

For the growth rate data set, as the growth rate decreases from 2.5 to 0.4 doublings per hour, a decrease in the concentration of most tRNAs leads to a slowdown of translational speed for nearly all codons (up to 2-fold, sustained response with major shift occurring between growth rates of 1.6 and 1.07 doublings per hour; Figure 2a). This data set does not include charged fractions of the entire pool, and therefore the influence of partial charging on codon rates is not captured here.

In contrast to the effectively uniform changes across all tRNAs for differing growth rates, the charging pattern during leucine starvation leads to differential rate changes that includes both slowing down and speeding up of codon rates (Figure 2b). The codons showing a pronounced speed up at all times are those coding for alanine, histidine and proline (up to 3.5-fold for Ala). As expected during leucine starvation, the largest decrease in availability is observed for the t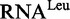 isoacceptors. This change is observed for all times, and the translational speed of all leucine codons decreases (up to 24-fold for the codons read by 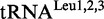 and up to 5-fold for the codons read by 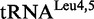).

In addition to these sustained responses, temporal changes were also observed during leucine starvation for some codons. Specifically, owing to the large decrease in charged fraction of most tRNAs measured at time point t = 17 min, the translation rates of the corresponding codons are temporarily slowed down (e.g. for Glu, Phe, Lys, Ile, Gln, Asn, Arg, and up to 4-fold for the Ile codon AUA).

### General features of the translational profiles

**Figure 3.**
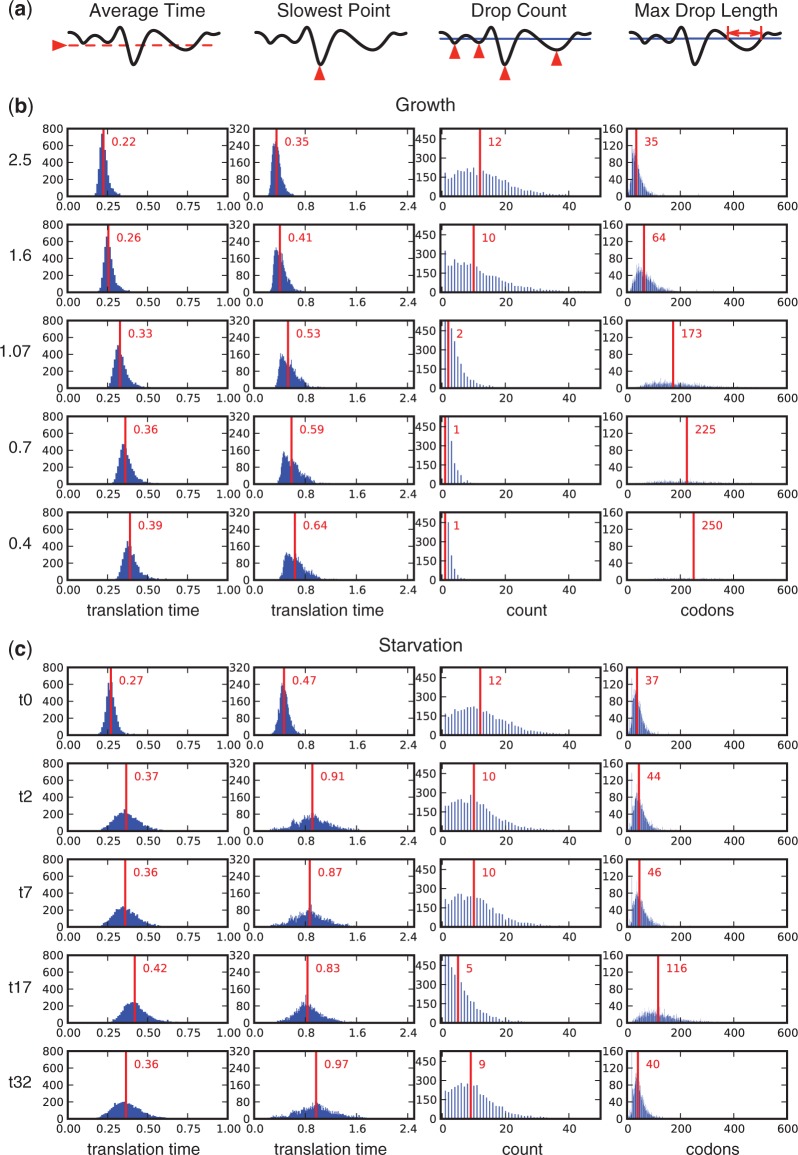
General features of translational profiles under differing conditions. (**a**) the four features that we analyzed for all profiles. Distributions of the features across the entire *E. coli* genome for (**b**) growth rate and (**c**) leucine starvation data sets. Red vertical lines and labels denote the median values of the distributions.

In addition to investigating the translational rates for individual codons, we attempted to capture the speed of ribosomal movement along an mRNA transcript. This depends on the choice and ordering of codons. To this end, we used a similar approach as in (10) and calculated averaged (smoothed) translational speed profiles for all *E. coli* genes under each growth and starvation condition (‘Materials and Methods’ section). These were then analyzed in terms of four general features as summarized in Figure 3a. As we find many profiles sensitive to the previously discussed changes in codon translation rates, the distributions of these features across the *E. coli* genome vary between different conditions (Figure 3b and c).

For the growth rate data, owing to translational profiles slowing down as the growth rate decreases, the distributions of average translation time and slowest translation time are shifted to higher values, and the skew toward longer times becomes more pronounced. If average time is regarded as a proxy for translational efficiency and the slowest point as a presumed rate limiting pausing site, these findings imply that under low growth conditions, a larger number of genes are translated less effectively.

The slowest points could, however, also link to attenuation sites required for co-translational folding. If minima below the threshold relate to the latter, we would expect a limited number of pronounced drops possibly corresponding to protein domains (10). However, as many profiles fluctuate close to the threshold, crossing it several times, we find a broad distribution of this feature. As the growth rate decreases, the number of drops gets concentrated and limited to lower values. At growth rates of 1.07, 0.7 and 0.4, there are almost no profiles that show >15, 10 or 5 drops, respectively. This is due to the general slowdown resulting in neighboring drops caused by small fluctuations near the threshold combining into single drops. As a consequence, the distribution of the maximal drop length becomes broadened toward higher values. We associate wider minima with an increased chance of a slowdown to actually occur during the stochastic elongation process. A similar effect we attribute to large numbers of neighboring drops and particularly deep minima. In addition, at lower growth rates, many profiles are slowed down so much that they remain entirely below the threshold. This leads to their exclusion from the analysis of the maximal drop length and explains the large increase in the bin counting profiles with a single drop.

For the leucine starvation data, when compared with time point t = 0 min, the distributions of average translation time and slowest point are broadened and shifted to higher values. Unlike under low growth rate conditions, these features remain more symmetrically distributed about their median value. For the drop count and maximal drop length, we observe a temporal response similar to the behavior described earlier in the text at time point t = 17 min, where several rates see a temporal decrease. In the distribution of the maximal drop length, a single bin is found with a high frequency. This corresponds to the window size used in profile smoothing and is the result of a single slow codon being able to pull the speed profile below the threshold for the entire window length. This peak is not present at time point t = 17 min where broader regions are shifted below the threshold.

Further analysis of the drop count and maximum drop length statistics was performed using thresholds calculated for each condition separately (Supplementary Figure S2). Although this did not allow for comparison between conditions (owing to a drop having a different minimum depth for each condition), it did enable us to see how general profile shapes, in terms of drops, varied in accordance with the average speed for that condition. For the growth rate data, we found that drop-related features were maintained across all growth rates (Supplementary Figure S2a). This is due to the fairly uniform rate changes ensuring that profiles maintain similar shapes (although at a lower overall average speed). In contrast, for the leucine starvation data, temporal changes are seen in the distributions that highlight large shifts in the drops present within the profiles (Supplementary Figure S2b). This is the result of the less uniform rate changes observed.

Under normal conditions, translational initiation is thought to be the rate-limiting step for expression of most natural genes (7). However, the shift toward slower translation times that we see under some conditions raises the possibility that some codons may become sufficiently slow to further limit translation. Under this scenario, there is the potential for ribosome queues to form. The susceptibility of a gene to this type of event is captured by the slowest point statistic (Figure 3). In terms of the genome distributions, we find that ribosomes queuing is more likely under leucine starvation, where larger rate changes enable particularly slow points to form. To investigate this potential further, we produced ranked lists of profiles in terms of their slowest point and analysed the functional enrichment of the 10% of genes containing the slowest points (Supplementary Data S3). We found significant enrichment of only a few common terms relating to the cell membrane and transport processes, with the number of enriched terms increasing for non-standard conditions.

### Sensitivity of translational profiles

In addition to analyzing general profile features, we also investigated to what extent individual profiles are sensitive to changes in tRNA availability. To do this, we compared profiles generated based on tRNA abundance under all growth and starvation conditions to a respective reference profile under standard conditions for each data set: growth rate of 2.5 doublings per hour and time point t = 0 min. A sensitivity value was then defined as the gene length normalized absolute differences between these two profiles (‘Materials and Methods’ section). Sensitivity analysis across the entire *E. coli* genome allowed for a ranking of the profiles to be performed, highlighting genes that were particularly sensitive or robust to fluctuating tRNA availability (Supplementary Data Set S1).

**Figure 4.**
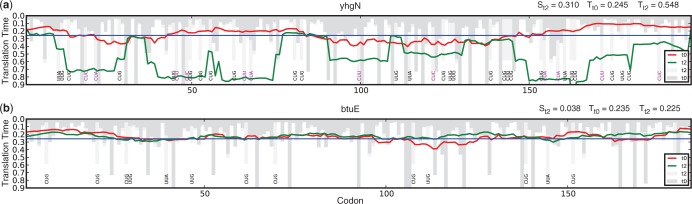
Changes to tRNA availability non-uniformly influence translational profiles across the genome. Comparison of two profiles taken from the (**a**) 10% most sensitive and (**b**) 10% least sensitive genes. These show the effect of changing rates for different leucine codons 2 min after leucine starvation. The high sensitivity of *yhgN* is mainly caused by the three low rate leucine codons CUU, CUC and CUA that vary greatly between these conditions. In comparison, these codons do not occur in the less sensitive sequence *btuE*, where only the fast CUG codon and the two less variable UUG and UUA codons are used. *yhgN* codes for an annotated non-essential inner membrane protein and *btuE* for a non-essential glutathione peroxidase. Red lines show the smoothed reference profiles before leucine starvation and green lines show the profiles 2 min after leucine starvation. The blue horizontal line indicates the threshold value used for the analysis of general profile features. Bars showing local codon speeds are colored in dark gray for reference condition and in light gray for starvation condition. To ensure the profile shape is clearly visible, some bars extend beyond the bottom of the plot. The annotation at the bottom shows all leucine codons, the three highly variable low rate leucine codons are shown in magenta and the faster and more robust codons are shown in black. 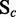 denotes the sensitivity value and 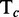 the average translation time under the respective condition *c*.

Depending on codon choices, the sensitivity to changes in tRNA abundance varies among *E. coli* genes. As the expected time on a codon is calculated assuming an exponential distribution, codons with tRNAs at low concentrations find that relatively small absolute fluctuations have a particular large effect on their speed. This is especially evident in the analysis of the different growth rates, which reveals that sequences containing several slow codons, and in particular the very slow AUA codon, have highly sensitive profiles. Hence, avoiding slow codons seems to be one way to render a profile more robust to translational rate variations (Figure 4 and Supplementary Figure S3).

Possibly in relation to this finding, sequences coding for essential genes were found to be more robust to variations in tRNA availability and generally avoided slow translating codons. In particular, the sensitivities of non-essential genes were found to be significantly higher when compared with essential genes under all conditions (*P* ≤ 4.6 × 10^−12^; Supplementary Table S3). This might be a partial effect of evolutionary pressure to efficiently produce essential genes, where avoiding slow codons leads to shorter average translation times. However, the importance of maintaining a robust profile shape has been recently demonstrated in experiments (5,6), where removal of possible translational pausing sites through overexpression of rare tRNAs leads to a decrease in protein solubility. Therefore, both overall speed and the need to maintain a profile shape for correct protein folding are likely to play roles in the fitness of underlying gene sequences as they evolve.

When considering the differential charging patterns during leucine starvation, another possible mechanism to maintain certain speed through a region was found. By using codons with opposing effects, i.e. some codons speed up, whereas other slow down, genes were able to maintain relatively robust profile shapes. This effect is clearly seen for the profiles of the histidine leader (*hisL*) sequence at time points t = 7 min and t = 17 min after leucine starvation (Supplementary Figure S5). At both time points, the two histidine codons are accelerated by approximately the same amount. However, at time point t = 17 min, the effect on the smoothed profile is partly compensated for by the slowdown of several preceding codons. Although it is difficult with only a limited number of conditions to evaluate if this mechanism is widely used, it does illustrate how multiple changes in tRNA availability can precisely control translational dynamics.

Another implication of the opposing rate changes during leucine starvation is a greater overall variability in profile shapes across the genome when compared with the different growth rate conditions. This is due to slower growth rates affecting all codon translation rates in a similar way, ensuring that qualitative features of the profiles are maintained. In contrast, the differential rate changes observed during leucine starvation provide a mechanism for significant changes in profile shapes.

**Figure 5.**
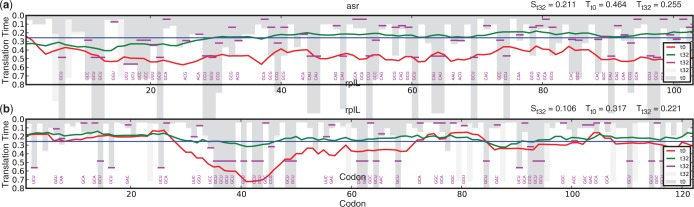
Selective charging of specific tRNAs under leucine starvation increases the translational speed of some genes. Shown are two sequences where the translation is accelerated during leucine starvation. (**a**) *asr* codes for an annotated non-essential acid shock-inducible protein and (**b**) *rplL* for an essential 50S ribosomal subunit protein. Red lines show the reference profiles at t = 0 min and green lines show the profiles t = 32 min after leucine starvation. The blue horizontal line indicates the threshold value used for the analysis of general profile features. Bars showing local codon speeds are colored in dark gray for reference condition and in light gray for rates that are decreased under starvation conditions. To ensure the profile shape is clearly visible, some bars extend beyond the bottom of the plot. Codons that show an increased rate under this condition are annotated in magenta, and the vertical lines denote the value of the accelerated speed. 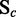 denotes the sensitivity value and 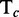 the average translation time under the respective condition *c*.

Although the majority of profiles saw a decrease in their average speed under all conditions, the increased speed of a few codons during leucine starvation did allow for accelerated translational profiles of some genes. Figure 5 shows an example of two genes that show speed up of translation at time point t = 32 min after leucine starvation owing to this mechanism. Further characterization of genes with large decrease in their average translation time found significant enrichment of structural constituents of the ribosome for time points t = 2, 7, 32 min after leucine starvation (*P *< 0.01; Supplementary Data Set S2).

### Functional enrichment of most and least sensitive genes

To investigate whether profile robustness or variability is particularly important within groups of genes related to specific functions, we considered the 10% most and least sensitive genes with respect to their biological context. We tested whether particular Gene Ontology (GO) terms were significantly enriched (*P* < 0.01) when compared with the entire *E. coli* genome (‘Materials and Methods’ section; Supplementary Data Set S2).

One would expect that, if shifts in tRNA availability is used as a control mechanism in *E. coli*, genes with regulatory roles or those only expressed under specific tRNA conditions (e.g. transiently expressed genes needed during a stress response) would be most sensitive to these changes. In contrast, genes playing an essential role or those with broad functionality needed at all times should display more robust profiles, ensuring changes in tRNA availability do not significantly affect protein production.

Supporting this hypothesis, analysis of the 10% most sensitive genes under different growth conditions revealed enrichment of genes involved in regulatory functions and specifically DNA-dependent regulation of transcription. Although the GO terms related to this are significantly enriched within the most sensitive genes at a growth rate of 1.6, the number of terms decreases as growth rates decreases. At a growth rate of 0.4, where changes compared with the standard growth rate 2.5 are most pronounced, only a few terms related to biofilm formation, pilus formation, lipopolysaccharide metabolism and defense response remain significantly enriched. This reduction in the number of enriched terms is likely due to larger changes in codon rates affecting a much broader range of genes. Thus, only rare terms in relation to the background set are retained under these conditions (Supplementary Data Set S2).

When analyzing the 10% least sensitive genes, the number of significant terms increases at lower growth rates (Supplementary Data Set S2). Owing to relatively small changes across a wide variety of different genes, only a few GO terms are enriched at a growth rate of 1.6. As the growth rate slows, larger rate changes occur and significant enrichment of GO terms related to metabolic functions (e.g. glycolysis, energy deviation/cellular respiration, nucleotide metabolism) or translational machinery (e.g. ribosomal subunits, tRNA aminoacylation) are observed.

Data for starvation conditions showed enrichment of similar terms related to metabolic and translational functions within the 10% least sensitive genes and enrichment of terms related to transcriptional regulation within the 10% most sensitive genes. In both cases, the number of significantly enriched terms was highest at time point t = 17 min, where a major shift in translation rate occurs for most codons.

### Increased sensitivity of regulatory leader sequences

Closer inspection of particularly sensitive genes highlighted an interesting regulatory mechanism that constitutes a way in which codon rates can significantly influence protein expression, even though they may not be rate limiting themselves.

In bacteria expression of amino acid biosynthesis operons are commonly regulated by transcriptional attenuation (25,26); a mechanism mediated by a leader region of the mRNA transcript preceding the structural genes of the operon. This leader mRNA can form two different and mutually exclusive stem-and-loop structures that either lead to premature termination of transcription or allow for transcription of the structural genes. The formation of a certain secondary structure is only possible if the required regions are not shielded by a translating ribosome. Hence, the decision for either of the structures depends on the efficiency with which the leader region is translated. This in turn is controlled by several regulatory codons that code for the amino acid related to the biosynthesis operon and where the speed of translation is highly sensitive to the abundance of cognate charged tRNAs.

We found the translational profiles of several leader sequences among the 10% genes that are most sensitive to measured changes is tRNA abundance. Specifically, the leader peptide *leuL* (27) of the leucine biosynthesis operon *leuLABCD* was found to be amongst the five most sensitive sequences at all times after leucine starvation. The deficiency of charged 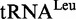 leads to a slower translation of four adjacent regulatory leucine codons, resulting in a large slowdown of the smoothed profile (Supplementary Figure S4a). Ribosome stalling at these codons supports the formation of the antiterminator and thus allows transcriptional read through to the structural genes.

A similar effect is observed within the leader sequences *ilvL* (28) and *ivbL* (29) controlling expression of two operons involved in valine and isoleucine biosynthesis (*ilvGEDA* and *ilvB*). During leucine starvation, we find a large slowdown at the regulatory Leu codons (Supplementary Figures S4b and c). Ribosome stalling at these codons prevents formation of the terminator and leads to increased expression of these operons.

The leader sequence *hisL* (30) of the histidine biosynthesis operon *hisLGDCBHAFI* is an example of a highly sensitive sequence with a more variable response to different conditions (Supplementary Figure S5). Under low growth rate conditions, translation of the *hisL* leader sequence slows down owing longer translation times at several codons including the seven regulatory histidine codons (Supplementary Figure S5a), allowing for transcription of the operon. In contrast, translation of the histidine codons is accelerated during leucine starvation (Supplementary Figure S5b), which allows for the terminator to form and transcription of the operon to halt. As discussed earlier, this effect is party compensated for at time point t = 17 min owing to a slowdown of other codons. It is not obvious why histidine would exhibit a more variable response. However, it may relate to the relatively rare usage across the genome leading to a less highly expressed set of biosynthesis genes and therefore reduced evolutionary pressure for strict regulation (Supplementary Table S4).

### Non-uniform and temporal changes across the genome

**Figure 6.**
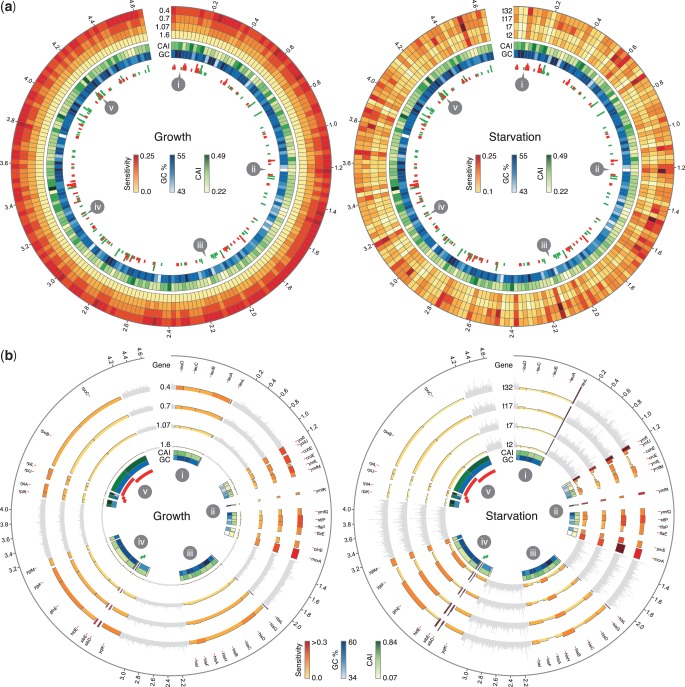
Genome-wide visualization of translational profile sensitivities. All plots display the entire *E. coli* genome and positions within the chromosome are indicated on the outer rim in Mbp. (**a**) Averaged sensitivities across the entire genome. The genome is split into 200 equal length portions, and averages are taken over genes that start within each region. Outer tracks show sensitivity heat maps (yellow = low, red = high) for differing conditions: (left) growth rates of 0.4, 0.7, 1.07, 1.6 and; (right) time points t = 32, 17, 7, 2 min after leucine starvation. The next two inner most heat maps show averaged general features of the genome. Specifically, the codon adaptation index (CAI) and GC%, with darker colors relating to higher values. Inner most tracks (displayed as bars) correspond to essential genes (red) and nucleotide sequences that are not translated (green). Each bar has a width the length of the gene it corresponds to, and bars are stacked in regions with high densities of essential genes or nucleotide sequences. (**b**) Gene-level sensitivities for the five highlighted regions marked on the upper plots. These relate to (i) the leucine biosynthesis operon *leuLABCD*; (ii) a region containing low GC% and CAI values; (iii) the histidine biosynthesis operon *hisLGDCBHAFI*; (iv) a region containing two untranslated nucleotide sequences; and (v) a region containing several essential genes related to the 50S ribosomal subunit and the RNA polymerase 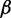 subunit. The height and color of the histogram tracks relate to the gene sensitivity. Inner most tracks match the upper plots displaying CAI, GC%, essential genes and nucleotide sequences. Highlighted regions are zoomed by a factor of 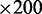 to ensure individual genes are visible. Non-highlighted regions are shaded in light gray.

With the aim of providing a more holistic view of how sensitivities vary across the entire genome, between conditions and times, and to discover possible links to other genomic features such as GC content and codon adaptation index (31), the Circos visualization tool was used (‘Materials and Methods’ section). Figure 6 shows genome-wide sensitivity for both data sets, and all conditions averaged over small regions of the genome and for several selected groups of individual genes.

When comparing between conditions, quantitative differences in the sensitivity profiles are revealed. The growth rate data set displays near uniform increases in sensitivity across the entire genome as the growth rate slows. This is most evident from the averaged heat maps (Figure 6a) that transition to deeper red values (increased sensitivity). As discussed previously, this relates to more uniform shifts found in all codon rates as growth rate decreases.

In contrast, after starvation, a far more specific response with temporal features is observed (Figure 6a). Particular regions in the genome are found to be highly sensitive or robust, and large differences in sensitivity are observed for specific time points. For example, at t = 17 min after leucine starvation, numerous localized points of high sensitivity arise. These specific responses are due to limited sets of codons displaying differential rate changes. Codon composition is known to vary between genes with differing functions (32), and therefore shifts in specific codon rates that might be overexpressed for genes with a particular function enable translational changes to affect certain functional subsets of genes that are often clustered in operon structures.

It is possible that such a localized shift in tRNA availability at t = 17 min after leucine starvation could be an experimental artifact. However, this seems unlikely owing to two main reasons. First, measurements of tRNA concentrations and charging are performed using a custom microarray that includes 18 replicates for each probe to help improve accuracy and reduce measurement errors (11). Second, although errors could still be introduced during sampling and sample preparation, if this were the case, then because a tRNA sample is prepared as one, we would expect all of the tRNAs to be affected in a non-specific manner. We do not find such broad differences in the tRNA availabilities, with the majority following a similar trend to measurements made at time points before (t = 7 min) and after (t = 32 min). Therefore, we believe that the temporal changes we see are accurately captured.

To better illustrate the variety of responses seen, five regions were selected containing genes of differing types; see highlighted points (i–v) in Figure 6a. Gene-level plots of sensitivity were then produced for both data sets and all conditions (Figure 6b).

Leader sequences were contained within regions (i) and (iii) for the leucine and histidine biosynthesis operons, respectively. Comparing between the data sets, the leucine leader (*leuL*) sees a large increase in its sensitivity under leucine starvation, whereas the structural genes downstream display virtually no change. This is expected as leucine biosynthesis capabilities are not necessarily required at slower growth rates but will be after a targeted starvation event. Moreover, as the structural genes for amino acid production form a core process for the cell, selection is likely to have acted to improve both efficiency and robustness of translation for these genes.

Conversely, the *hisL* sequence sees elevated sensitivity under virtually all conditions and a more variable response after leucine starvation. However, once again, the structural genes of the pathway display more robust profiles, although to a lesser extent than for leucine biosynthesis and likely due to histidines much rarer usage (Supplementary Table S4).

Genes with low CAI and GC% values were contained within region (ii). These displayed increased sensitivity and a much greater variability in the size of the response seen when compared with other regions. For example, *cohE* (a predicted repressor protein) only sees increased sensitivity at slower growth rates, whereas *ymfI* (an uncharacterized protein) displays strong sensitivity under all conditions.

The range of CAI and GC% values observed in this region revealed a link between these sequence-based measures and profile sensitivity. Specifically, when comparing CAI to sensitivity values under all conditions, highly significant correlations were found (Spearman’s rank, *P* 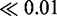; Supplementary Table S5). Extending this analysis to GC% also yielded significant correlations. However, the goodness of fit for these was much less, and in several cases, it was extremely low (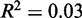 for t = 2, 7 and 32 min after leucine starvation).

The link between CAI and sensitivity likely stems from the fact that the CAI relies on a set of highly expressed genes for calculation of codon biases (31). As highly expressed genes are likely to have experienced strong evolutionary pressure to maintain robust profiles under normal conditions, biases contained within these genes will become captured by the CAI value. Supporting this hypothesis, we found that when comparing with conditions where rates deviated more significantly from standard conditions, i.e. after leucine starvation, the goodness of fit for the correlations drops from an average 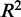 value of 0.445 between growth rate conditions, to 0.362 under leucine starvation (Supplementary Table S5).

Region (iv) contained two untranslated nucleotide sequences (*sibD* and *sibE*) that we would assume are uninfluenced by tRNA availability. These genes both displayed increased sensitivity under all conditions. Assuming there exists no evolutionary pressure on these sequences for reduced sensitivity, we tested whether the sensitivity of untranslated nucleotide sequences was higher than for protein-coding sequences (Supplementary Table S3). As expected, significant differences were observed for growth rates of 1.07, 0.7 and 0.4 doublings per hour and at 17 min after leucine starvation (*P*-value < 2.2 × 10^−16^; Supplementary Table S3).

Lastly, region (v) contained a set of essential genes that form structural constituents of the ribosome and RNA polymerase. As would be expected for genes expressed at high levels and at all times, these display low sensitivities to all conditions and vary together in a highly uniform way.

## DISCUSSION

In this work, we developed a computational workflow for estimating codon translation rates based on tRNA availability. We used this to generate profiles that capture the translational speed along a transcript. By considering experimentally measured tRNA availabilities under differing growth rates and for time points after leucine starvation, we were able to reveal several interesting ways in which translation can be affected across the *E. coli* genome.

When analyzing general features of the profile shapes, we found that for both conditions, a slowdown in average translation speed occurred. This increased with slower growth rates and remained fixed after an initial slowdown during leucine starvation. These differences were attributed to growth rate having a relatively uniform effect across all codon rates, whereas leucine starvation displayed more differential changes, with the majority of codons decreasing, but some increasing their rate.

By assessing the sensitivity of the translational profiles under different conditions, we found that as with the average translational speed, gene sensitivity saw a more uniform increase for decreasing growth rates, and more differential changes during leucine starvation. However, careful examination of the profiles did uncover the exclusion of rare codons and the use of codons with opposing rate changes to improve the robustness of some profiles.

Ranking genes in terms of their sensitivity to shifts in tRNA availability showed that robust profiles related to genes that were essential or involved in core functions, whereas highly sensitive profiles were linked to genes with more specific regulatory roles or which play specific roles during a stress response. Furthermore, many of the most sensitive genes were those where translational control was known to play a major role. Specifically, leader peptide sequences for amino acid synthesis operons were found to be highly sensitive. These allow for small rate changes in specific codons to effectively turn on or off the transcription of the structural genes in the associated amino acid biosynthesis pathways.

For most natural translational processes, ribosome initiation is believed to be the rate-limiting step (7), and therefore the codon translation rates presented here may not significantly contribute to protein levels themselves. However, as we show, these rates can have indirect links to protein expression via their impact on various features of the translational speed profiles. Translational pausing caused by clusters of slow codons is regarded as crucial in some cases to ensure correct protein folding (4,10) and enhance solubility (5,6), whereas stalls located near the start of a transcript can have adverse effects of expression by inhibiting ribosome initiation. The complexity and often conflicting influences of translational rate changes make our tool valuable for understanding and predicting such effects.

An interesting future direction that builds on this work is to incorporate a fuller description of the dynamical changes to tRNA and ribosome pools during a change in conditions. Here, we extend previous studies by allowing for measured shifts in tRNA availability but neglect possible transient changes as this takes place. This could be particularly important when considering the overexpression of a recombinant protein, where a specific codon composition might lead to the depletion of certain charged tRNA pools (33) or under amino-acid limited growth conditions that have been shown to lead to specific charging patterns (34). Moreover, it has recently been found that codon composition of the entire transcriptome, and thus tRNA demand, changes dynamically under environmental stress (35).

Further refinement of our model could also be made through incorporation of a wider range of potential post-transcriptional modifications to tRNAs (13,14). These modifications come in many different forms. However, for our analysis, the most relevant are uridine methyl-transferases that alter the wobble position 34 of certain tRNAs, causing changes in the recognition and sensitivity of particular codon-anticodon pairings. These can be incorporated by updating the rules for the conversion from tRNA availability to codon translation times.

The workflow developed here has solely been used for understanding how translation of endogenous sequences behaves under varying conditions, attempting to reverse engineering how translational control is used by *E. coli*. There is also the possibility to use the same workflow in a forward engineering mode. Under this scenario, rather than providing sequences and tRNA availabilities, a required profile shape would instead be given. By using the workflow within an optimization framework, it is then possible to design genes with the rational use of slow and fast codons that introduce required local translational features. This approach would allow for future investigations into the possible effect that co-translational folding can have on synthetic genes (e.g. to assist in the correct domain structures being formed) and in the design of synthetic leader sequences that, similar to bacterial regulation of amino acid synthesis operons, enable a way of significantly controlling gene expression through tRNA availability and transcriptional attenuation (25,26).

To test the feasibility of this approach, preliminary tests were run on a library of synthetic genes variants (16). All genes coded for the same amino acid sequence (a single-chain antibody fragment), but each included differing synonymous codon usage. Comparing variant profiles under leucine starvation conditions showed a large variability in the types of profile produced (Supplementary Figure S6). Although further analysis was outside the scope of this work, these findings did highlight the potential flexibility for modulating profile shape to particular conditions while maintaining a fixed protein sequence.

Finally, although this study further strengthens the important and diverse roles that tRNA availability plays in controlling translational processes (4–6,20,25,36,37), there remains a significant lack of data sets capturing the temporal changes of both concentrations and charging of individual tRNAs under different stress conditions. These will be essential to broaden our understanding of tRNA-mediated translational control across the genome and for the potential application of this knowledge to synthetic gene design (38). Furthermore, by comparing these model predictions with new high-resolution ribosome profiling techniques (39), we have the opportunity to validate these approaches at the genome-scale.

## SUPPLEMENTARY DATA

Supplementary Data are available at NAR Online.

## FUNDING

DSM student internship (to S.W.); European Commission funded Marie-Curie Actions Initial Training Network for Integrated Cellular Homeostasis (NICHE) project [289384 to T.E.G.]. Funding for open access charge: Open-access publication charges will be covered by DSM.

*Conflict of interest statement.* None declared.

## Supplementary Material

Supplementary Data
