# Supplementary material for: Translational sensitivity of the Escherichia coli genome to fluctuating tRNA availability
Source: Nucleic Acids Res. 2013 Jul 10;41(17):8021–33. doi: 10.1093/nar/gkt602 (PMC3783181; doi:10.1093/nar/gkt602)
Supplement: Supplementary Data [file supp_41_17_8021__index.html]

Translational sensitivity of the Escherichia coli genome to fluctuating tRNA availability — Translational sensitivity of the Escherichia coli genome to fluctuating tRNA availability — Supplementary Data 

# Translational sensitivity of the *Escherichia coli* genome to fluctuating tRNA availability

## 

files

**Files in this Data Supplement:**

- Supplementary Data - zip file
