# Supplementary material for: Translational sensitivity of the Escherichia coli genome to fluctuating tRNA availability
Source: Nucleic Acids Res. 2013 Jul 10;41(17):8021–33. doi: 10.1093/nar/gkt602 (PMC3783181; doi:10.1093/nar/gkt602)
Supplement: Supplementary Data [file supp_gkt602_nar-01211-z-2013-File003.zip › Translational_Sensitivity-SI.pdf]

Supplementary Information: Translational sensitivity of the Escherichia coli genome to fluctuating tRNA availability

Sibylle Wohlgemuth†, Thomas E. Gorochowski*, and Johannes A. Roubos

DSM Biotechnology Center, P.O. Box 1, 2600 MA Delft, The Netherlands

†Present Address: Institute for Computational Science, ETH Zurich, 8092 Zurich, Switzerland

Contents

Supplementary Figures **2**

Figure S1 Possible codon-anticodon interactions according to Crick's Wobble rules 2

Figure S2 Drop count and maximum drop length statistics of translational profiles 3

Figure S3 Presence of 'slow' codons increases translational profile sensitivity 4

Figure S4 Translational profiles of leader peptides of amino acid biosynthetic operons 5

Figure S5 Comparison of hisL leader peptide under four different conditions 6

Figure S6 Synonymous codon choice can have a large effect on translational profiles 7

Supplementary Tables **8**

Table S1 s-values of different wobble pairings 8

Table S2 Calculation of adaptiveness values 9

Table S3 Sensitivity analysis of essential genes and nucleotide sequences 10

Table S4 Amino acid usage in the E. coli genome 11

Table S5 Spearman's rank correlation analysis of translational profile sensitvity 12

References **13**

*To whom correspondence should be addressed. Email: thomas.gorochowski@dsm.com

Supplementary Figures

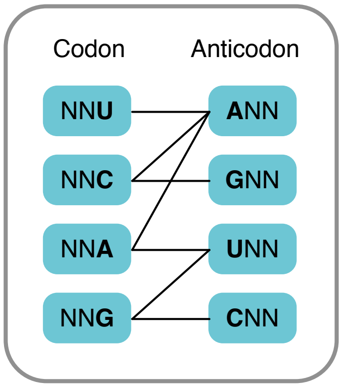

Figure S1: Possible codon-anticodon interactions according to Crick's Wobble rules [1]. The first two positions remain fixed, while the last position allows for some wobble interactions with differing affinities.

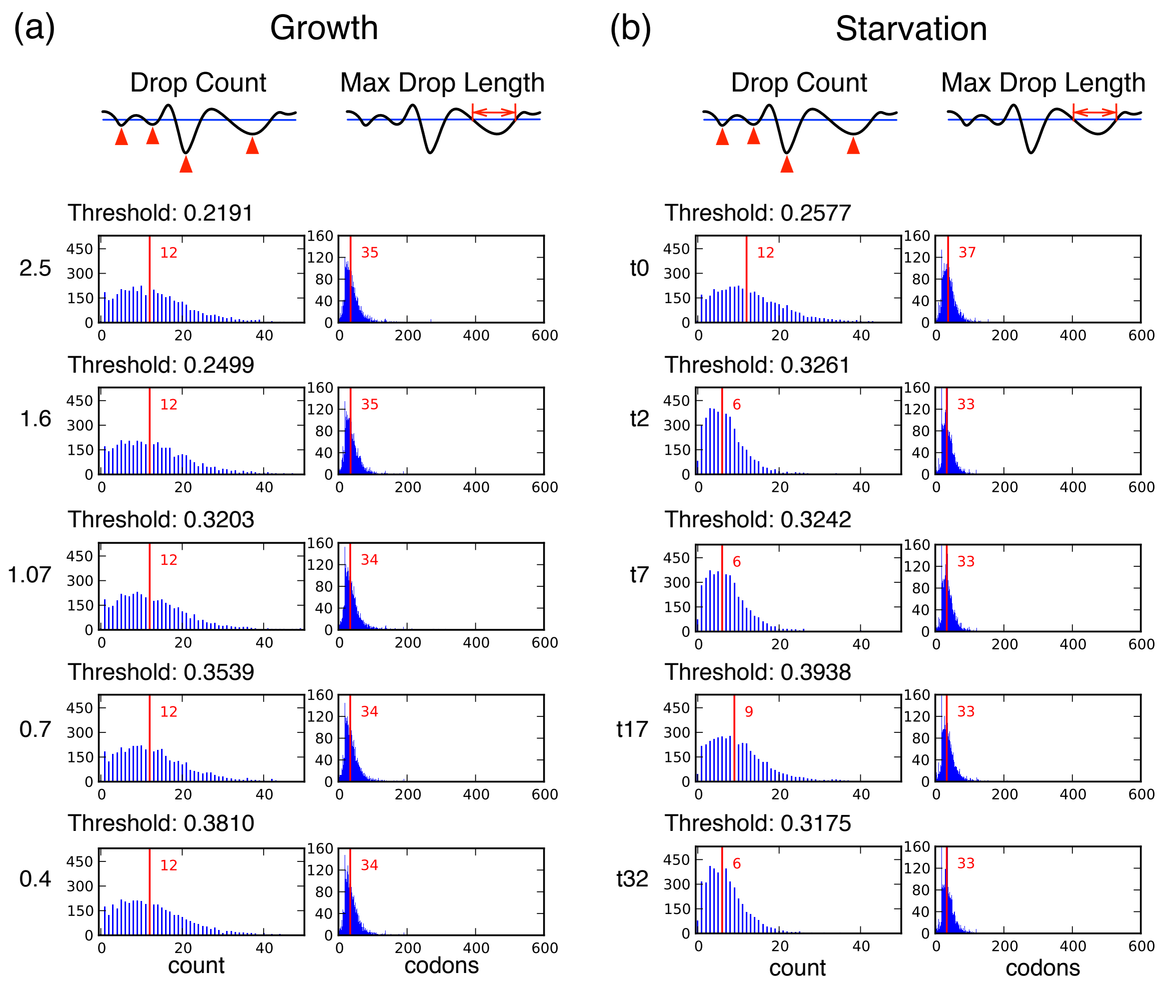

Figure S2: Drop count and maximum drop length statistics of translational profiles under differing conditions. Distributions of the features across the entire *E. coli* genome for **(a)** growth rate and **(b)** leucine starvation data sets. Red vertical lines and labels denote the median values of the distributions. Thresholds were individually calculated for each condition separately and are shown for each pair of plots (see Materials and Methods section in the main text for further details).

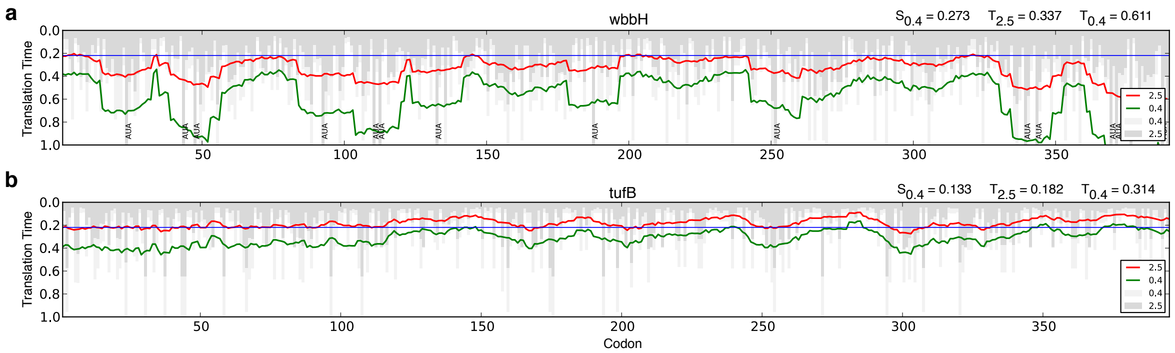

Figure S3: Presence of 'slow' codons increases translational profile sensitivity. Compared are two profiles among the 10% most **(a)** or least **(b)** sensitive genes respectively. The highly sensitive sequence shows a much rougher profile and a higher average translation time due to several slow codons, in particular the very slow AUA codon. *wbbH* codes for the non-essential O-antigen polymerase and *tufB* for the essential elongation factor Tu. Red lines show the smoothed reference profiles under standard growth rate 2.5 and green lines show the profiles at growth rate 0.4. The blue horizontal line indicates the threshold value used for the analysis of general profile features. Bars showing local codon speeds are colored in dark gray for reference condition and in light gray for decreased growth rate. Note that to ensure the profile shape is clearly visible, some bars extend beyond the bottom of the plot. The annotation at the bottom shows all occurrences of the slow AUA codon. S_c denotes the sensitivity value and T_c the average translation time under the respective condition c.

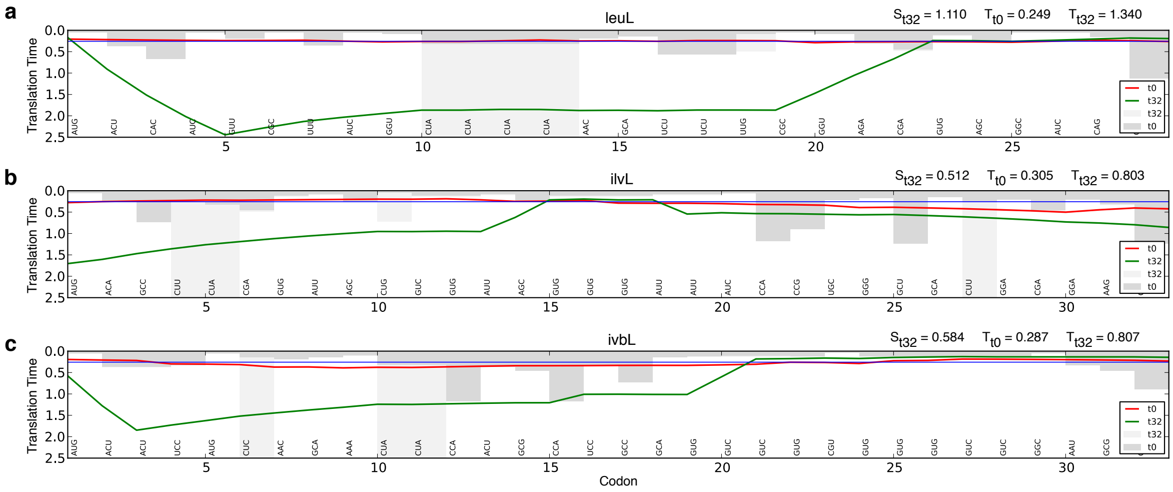

Figure S4: Translational profiles of three leader peptides of amino acid biosynthetic operons. Shown are the profiles of the three leader peptides for the leucine **(a)** and isoleucine and valine biosynthetic operons **(b, c)**. In each case a large slowdown is formed during leucine starvation. Red lines show the reference profiles at time 0 min and green lines show the profiles 32 min after leucine starvation. The blue horizontal line indicates the threshold value used for the analysis of general profile features. Bars showing local codon speeds are colored in dark gray for reference condition and in light gray for rates that decrease under starvation conditions. Note that to ensure the profile shape is clearly visible, some bars extend beyond the bottom of the plot. S_c denotes the sensitivity value and T_c the average translation time under the respective condition c.

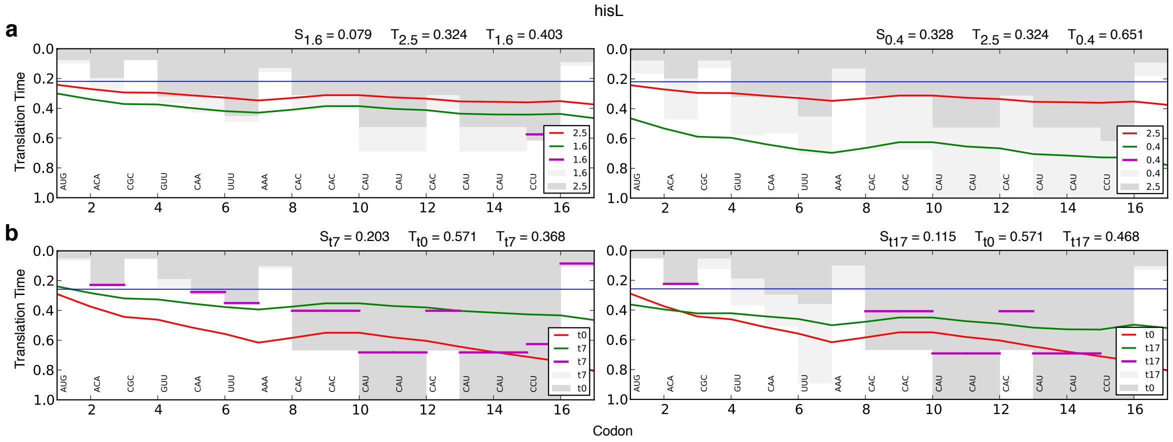

Figure S5: Comparison of the profile for the *hisL* leader peptide under four different conditions. Under low growth rate conditions the profile is shifted to longer translation times **(a)**. In contrast, during leucine starvation translation of the sequence is accelerated **(b)**. Interestingly, the speed up is less pronounced 17 min after leucine starvation even though the acceleration of the histidine codon rate is nearly the same as after 7 min. This is caused by several preceding codons that due to their slowdown have a compensating effect. Red lines show the reference profiles at time 0 min or standard growth rate 2.5 and green lines show the profiles 7 min after leucine starvation or at growth rate 0.4. The blue horizontal line indicates the threshold value used for the analysis of general profile features. Bars showing local codon speeds are colored in dark gray for reference condition and in light gray for rates that decrease under the respective condition. Note that to ensure the profile shape is clearly visible, some bars extend beyond the bottom of the plot. Horizontal magenta lines indicate the value of increasing rates. S_c denotes the sensitivity value and T_c the average translation time under the respective condition c.

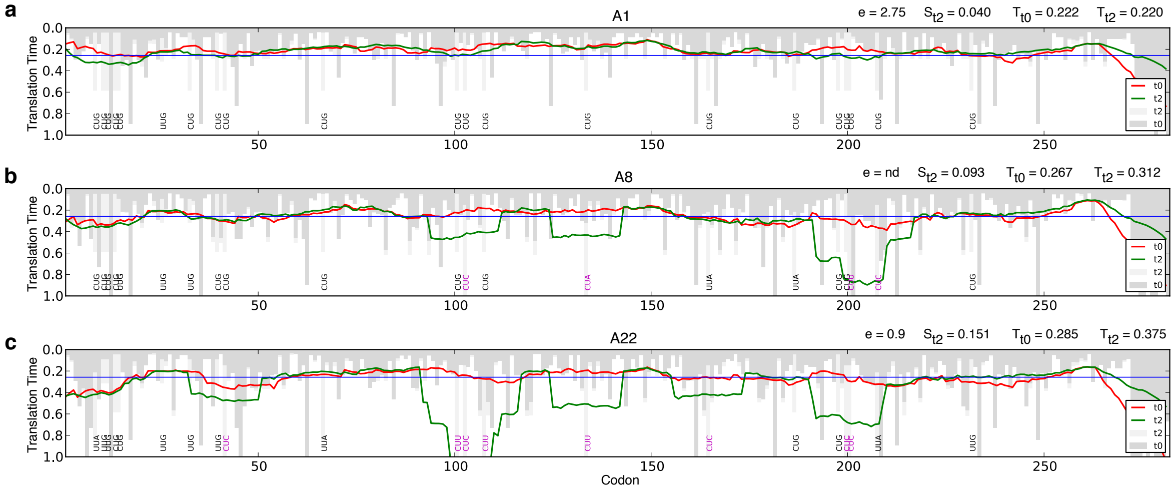

Figure S6: Synonymous codon choice can have a large effect on translational profiles. Shown are the profiles of three synthetic variants of a single chain antibody where the choice of synonymous leucine codons shown in magenta has a big impact on the profile sensitivity. Red lines show the reference profiles at time 0 min or and green lines show the profiles 2 min after leucine starvation. The blue horizontal line indicates the threshold value used for the analysis of general profile features. Bars showing local codon speeds are colored in dark gray for reference condition and in light gray for rates that decrease under the respective condition. Note that to ensure the profile shape is clearly visible, some bars extend beyond the bottom of the plot. S_c denotes the sensitivity value and T_c the average translation time under the respective condition c and e the relative expression as measured by Welch *et al.* [2].

Supplementary Tables

Table S1: s-values for the efficiency of different wobble pairings. I, inosine; L, lysidine (recognition of AUA-codon by LAU-anticodon in prokaryotes).

**Anticodon:codon**	I:U	G:C	U:A	C:G	G:U	A:C	A:A	U:G	L:A
**s-value**	0.0	0.0	0.0	0.0	0.41	0.28	0.9999	0.68	0.89

Table S2: Calculation of adaptiveness values [3].

n	**Anticodon**	**Codon**	W
i	INN	NNU	$(1 - s_{\mathrm{I:U}})\mathrm{tRNA}_i + (1 - s_{\mathrm{G:U}})\mathrm{tRNA}_{i+1}$
$i+1$	GNN	NNC	$(1 - s_{\mathrm{G:C}})\mathrm{tRNA}_{i+1} + (1 - s_{\mathrm{I:C}})\mathrm{tRNA}_i$
$i+2$	UNN	NNA	$(1 - s_{\mathrm{U:A}})\mathrm{tRNA}_{i+2} + (1 - s_{\mathrm{I:A}})\mathrm{tRNA}_i$
$i+3$	CNN	NNG	$(1 - s_{\mathrm{C:G}})\mathrm{tRNA}_{i+3} + (1 - s_{\mathrm{U:G}})\mathrm{tRNA}_{i+2}$

Table S3: Sensitivity analysis of essential genes and nucleotide sequences. Essential genes were compared to non-essential genes to see if there was a significantly reduced sensitivity. Non-protein coding nucleotide sequences were compared to protein coding genes to see if there was a significiant increase in sensitivity. A non-parametric Mann-Whitney-Wilcoxon test was performed using R version 2.15.3 and the p-values are reported in the table. All sensitivity values were generated in relation to standard reference conditions: 2.5 doublings per hour for growth rate data, and t = 0 min after leucine starvation.

**Sensitivity to Reference Condition**	**Essential vs Non-Essential**	**Protein Coding vs Nucleotides**
Growth 0.4 doublings per hour	$<$ 2.2E-16	$<$ 2.2E-16
Growth 0.7 doublings per hour	$<$ 2.2E-16	$<$ 2.2E-16
Growth 1.07 doublings per hour	2.20E-16	$<$ 2.2E-16
Growth 1.6 doublings per hour	4.60E-12	9.92E-01
Starvation t = 2 min	1.13E-14	1.66E-01
Starvation t = 7 min	4.01E-15	2.78E-01
Starvation t = 17 min	$<$ 2.2E-16	$<$ 2.2E-16
Starvation t = 12 min	1.29E-15	1.31E-01

Table S4: Amino acid usage in the *E. coli* genome.

**Amino Acid**		**Count**	**Percentage**
C	Cys	15671	1.18
W	Trp	20426	1.54
H	His	30093	2.27
M	Met	36970	2.78
Y	Tyr	37402	2.82
F	Phe	51560	3.88
N	Asn	51615	3.89
K	Lys	58291	4.39
Q	Gln	58902	4.44
P	Pro	59292	4.47
D	Asp	67772	5.10
T	Thr	71401	5.38
R	Arg	74238	5.59
E	Glu	76507	5.76
S	Ser	76906	5.79
I	Ile	79401	5.98
V	Val	94532	7.12
G	Gly	98153	7.39
A	Ala	126479	9.53
L	Leu	141977	10.69

Table S5: Spearman's rank correlation analysis of genome features to translational profile sensitvity. All sensitivity values were generated in relation to standard reference conditions: 2.5 doublings per hour for growth rate data, and t = 0 min after leucine starvation. Exact p-values are provided where possible. Analysis was carried out using TIBCO Spotfire version 4.5.

Variable	Response: Sensitivity to Reference Condition	R^2	R	p-value
CAI	Growth 0.4 doublings per hour	0.49	-0.70	$<$ 3.51E-312
	Growth 0.7 doublings per hour	0.46	-0.68	$<$ 3.51E-312
	Growth 1.07 doublings per hour	0.51	-0.72	$<$ 3.51E-312
	Growth 1.6 doublings per hour	0.32	-0.56	$<$ 3.51E-312
	Starvation t = 2 min	0.29	-0.54	3.51E-312
	Starvation t = 7 min	0.31	-0.56	$<$ 3.51E-312
	Starvation t = 17 min	0.54	-0.73	$<$ 3.51E-312
	Starvation t = 12 min	0.31	-0.56	$<$ 3.51E-312
GC%	Growth 0.4 doublings per hour	0.20	-0.45	7.41E-208
	Growth 0.7 doublings per hour	0.20	-0.44	3.24E-201
	Growth 1.07 doublings per hour	0.18	-0.42	3.29E-178
	Growth 1.6 doublings per hour	0.21	-0.46	5.80E-215
	Starvation t = 2 min	0.03	-0.16	1.95E-25
	Starvation t = 7 min	0.03	-0.17	7.54E-29
	Starvation t = 17 min	0.18	-0.42	7.27E-176
	Starvation t = 32 min	0.03	-0.17	5.48E-28

References

[1] Crick, F.H.C. (1966) Codon-anticodon pairing: the wobble hypothesis. *Journal of Molecular Biology*, **19**, 548–555.

[2] Welch, M., Govindarajan, S., Ness, J.E., Villalobos, A., Gurney, A., Minshull, J. and Gustafsson, C. (2009) Design parameters to control synthetic gene expression in *Escherichia coli*. *PLoS ONE*, **4**, e7002.

[3] dos Reis, M., Savva, R. and Wernisch, L. (2004) Solving the riddle of codon usage preferences: a test for translational selection. *Nucleic Acids Research*, **32**, 5036–5044.
